# The histological prevalence of prostatitis at Potchefstroom Hospital: a cross-sectional study

**DOI:** 10.11604/pamj.2024.47.8.40583

**Published:** 2024-01-09

**Authors:** Mwila Prince Kasongo, Essame Philippa, Witts-Hewinson Fabienne, Yimbo Marylyne, Mohamed Behnazir

**Affiliations:** 1 Department of Surgery, Potchefstroom Hospital, North West Province, Potchefstroom, South Africa,; 2 Department of Anaesthesia, Potchefstroom Hospital, North West Province, Potchefstroom, South Africa,; 3 Department of Orthopaedics, Potchefstroom Hospital, North West Province, Potchefstroom, South Africa

**Keywords:** Prostatitis, prevalence, histology, prostate biopsy

## Abstract

**Introduction:**

prostatitis is defined as a clinical condition caused by acute or chronic infectious diseases, chronic pelvic pain syndrome, or asymptomatic inflammation of the prostate gland. We conducted a study to determine the prevalence of histological prostatitis in patients with prostatic diseases at Potchefstroom Hospital.

**Methods:**

we conducted a cross-sectional study based on the review of histology report from 1^st^ January 2015 to 31^st^ December 2019 coupled to clinical information of patients. A total of 362 cases with complete histopathology report were included in the study. Chi-square and Fisher exact test were used to test statistical significance with a p-value of 0.05 deemed to be significant.

**Results:**

over a total number of 362 patients, the mean age was 69.82±7.9 years. The overall prevalence of prostatitis on histology was 158 (43.65%). Chronic prostatitis was predominant and commonly associated with BPH or PCa in 142 cases (39.23%) on histopathology report, while acute prostatitis was found in 16 patients (4.42%). We further found prostate cancer in 178 patients (49.17%), BPH in 163 (45.03%). The study shows that 41 cases (23.3%) of prostate cancer were associated with prostatitis, and 96 cases (58.9%) of BPH were also associated with prostatitis on histology. The difference was statistically significant p < 0.001. The study revealed that in BPH with prostatitis the median PSA was 15 ng/ml (IQR 9-24), while in BPH without prostatitis the median PSA was 11ng/ml (IQR 7-16). (p < 0.017). This means that prostatitis increases PSA in patients with BPH. However, the presence of prostatitis did not significantly influence the PSA value in patients with prostate cancer.

**Conclusion:**

this study showed that close to half of the histology examined had signs of prostatitis. Chronic prostatitis was more frequent on histology done in our sample. BPH was strongly associated with prostatitis. Prostatitis contributed to a higher PSA values in patients with BPH and did not influence the PSA value in PCa.

## Introduction

Prostatitis can simply be defined as the inflammation of the prostate gland. However, the current definition includes several clinical presentations. It is defined as a clinical condition caused by acute or chronic infectious diseases, chronic pelvic pain syndrome, or asymptomatic inflammation [1,2]. The prostate is a walnut-sized gland surrounding the urethra, situated just below the bladder. The prostate's primary function is to produce prostatic fluid and it can be affected by numerous pathologies, like inflammation and various consequences [3].

Prostatitis has many aetiologies; it can be either infectious or non-infectious [1,3-5]. Bacterial infection is sometimes implicated, but it is often due to non-bacterial causes such as chronic inflammation, autoimmune diseases and endocrine diseases [1,4,6]. Prostatitis is very common. It is estimated that up to 25% of men are diagnosed with prostatitis in their lifetime [7]. Furthermore, it is the most common cause of chronic pelvic pain in elderly men [6,8]. Chronic prostatitis can be difficult to diagnose and treat as it has a high rate of recurrence and a wide range of differential diagnoses. Therefore, chronic pelvic pain, the hallmark clinical sign of prostatitis, can affect quality of life negatively [6,9-12].

The National Institute of Health's classification system has been adopted internationally as one of the best ways to classify prostatitis. It distinguishes four categories: acute bacterial prostatitis, chronic bacterial prostatitis, chronic prostatitis or chronic pelvic pain syndrome (inflammatory and non-inflammatory subtypes), and lastly asymptomatic inflammatory prostatitis [1,13-15]. It is important to understand the relationship between prostatitis and other prostatic conditions, the value of prostatitis in the workup of prostatic diseases, as well as its influence on prostate specific antigen (PSA) value [16]. Prostatitis can be a stand-alone histological diagnosis or it can coexist with other prostatic diseases. Previous studies conducted in other countries and parts of South Africa have shown an association between histological prostatitis and benign prostatic hyperplasia (BPH), as well as histological prostatitis and cancer of the prostate (PCa) [13,16-18]. In South Africa, with a high volume of the population attending public hospitals, patients with prostatic diseases sometimes live with indwelling catheters for several months before definitive surgical management. This situation predisposes patients to prostatic and urinary infections [16,17].

The aim of this study is to determine the prevalence of histological prostatitis in patients with prostatic diseases at Potchefstroom Hospital during the study period, to evaluate the association between prostatitis and BPH, prostatitis and prostate cancer, and to analyse the impact of prostatitis on the PSA value in our setting.

## Methods

**Definition:** the National Health Insurance (NHI) defines prostatitis as a clinical condition caused by acute or chronic infectious diseases, chronic pelvic pain syndrome, or asymptomatic inflammation. However, in this study we reported acute or chronic prostatitis based on the conclusion of the histopathological report (Microscopic features of acute or chronic inflammation).

**Study design:** we conducted a retrospective cross-sectional study at Potchefstroom Hospital based on the review of histology reports from 1^st^ January 2015 to 31^st^ December 2019, coupled with clinical information of patients.

**Study setting:** Potchefstroom Hospital is a regional hospital located in North West province, South Africa, that offers basic urology services with support from the Urology Department at Klerksdorp Hospital. The department is run by two general surgeons who supervise four medical officers and at least four medical interns each year. Patients with urology complaints are seen at the department, all ancillary investigations and imaging are done in consultation with the urology department from the tertiary hospital situated at about 50 kilometres in Klerksdorp.

**Inclusion and exclusion criteria:** we conducted a retrospective review of patients with signs and symptoms of acute or chronic prostatitis. However, we only included patients with histopathology results of the prostate. All other patients, especially younger ones who presented with signs and symptoms of prostatitis but did not have an indication of a prostate biopsy were not included. Data were extracted from patient's records (Age, race, PSA) and histology reports (Prostatitis, BPH, PCa).

**Sample size:** from a total of 405 files of patients who underwent a prostate biopsy during the study period, 43 files with missing histopathology results were excluded. As a result, we retained the 362 files with complete information as our convenient sample size. With a prevalence of prostatitis on histology estimated at 40%, the confidence interval of 95% and 5% precision, the minimum sample size was estimated at 245 participants.

**Biopsy technique and risk of bias:** a trans-perineal approach was used to take biopsies in all four quadrants. The indication for a prostate biopsy was a presence of lower urinary tract symptoms with a PSA equal or above 4 ng/ml. An average of 10 to 12 core biopsies was taken under regional anaesthesia in theatre (Spinal) for all cases included in this study. Over the study period, different operators (community service doctors, junior and senior medical officers) collected the biopsies which were also reported by different pathologists (Specialists). This is considered as a potential source of bias.

**Specimen handling:** once the biopsy specimens were collected, they were immediately kept in the formalin solution and routinely transported to the National Health Laboratory Service at the end of the theatre list for analysis.

**Data analysis, reporting:** data analysis was performed with STATA 15.1 (Startacorp, college, Texas 7784 USA). Normally distributed continuous variables were reported as means with standard deviation, skewed variables as median and interquartile range, and categorical variables as frequency or percentage. A Chi-square test was used to examine the association between prostatitis with BPH and prostatitis with prostate cancer. A Fischer's exact test was used to study the association between prostatitis and PSA in patients with BPH and to examine the association between prostatitis and PSA in patients with prostate cancer. A p-value of less than 0.5 was deemed statistically significant. We reported the overall prevalence of acute and chronic prostatitis on the histology sample, and we studied the association between prostatitis with BPH and prostatitis with PCa.

**Ethics considerations:** the ethical approval was granted by the Health Research Ethics Committee (HREC Nr. S21/04/069) and the goodwill letter by the North West province Department of Health and the hospital's ethics committee. Informed consent was waived as the study was retrospective and based on the audit of existing records.

## Results

A total of 362 biopsy samples were examined. The overall prevalence of prostatitis in histology was 43.65% (158/362). Chronic prostatitis was predominant and commonly associated with BPH or PCa (39.23%) on histopathology reports (142/362), while acute prostatitis was found in 4.42% (16/362).

The overall mean age of participants was 69.82±7.9 years. Moreover, the mean age of patients with prostatitis in this group was 70.79±7.69 years with the age group of 61-70 years being the most represented 41.71% (151/362), followed by the 71-80 years 37.29% (135/362). The majority of participants were African 75.69% (274/362), followed by Caucasian 12.15% (44/362) and mixed race 11.88% (43/362) (Table 1).

**Table 1:** demographics and prostatitis prevalence based on histology

Population group	Frequency and %
African	274 (75.69%)
Caucasian	44 (12.15%)
Mixed race	43 (11.88%)
Asian	1 (0.28%)
Total	362
**Age groups**	**Frequency and %**
50-60	46 (12.71%)
61-70	151 (41.71%)
71-80	135 (37.29%)
81-90	27 (7.46%)
90-100	3 (0.83%)
Total	362
**Prostatitis prevalence**	**Frequency**
Chronic prostatitis	142/362 (39.23%)
Acute prostatitis	16/362 (4.42%)
Total	158/362 (43.65%)

Table 2 shows that from all the prostate cancer cases, 23.3% (41/178) were associated with prostatitis, while 58.9% (96/163) of BPH were also associated with prostatitis on histology. The Chi-square test showed (Table 2) that there is statistically significant difference p < 0.001 in the number of prostatitis cases associated with BPH compared to the number of prostatitis cases in patients with prostate cancer. Our analysis reveals that prostatitis is more likely to be associated with BPH than with PCa.

**Table 2:** association between prostatitis and BPH, prostatitis and PCa

	BPH	PCa	Total	p-value
Prostatitis	96 (58.9%)	41 (23%)	137	< 0.001
No prostatitis	67 (41.1%)	137 (77%)	204	
Total	163	178	341	

Table 3 displays the relationship between BPH, PCa, prostatitis and PSA. As expected, 25.77% (42/163) of patients with BPH had a PSA above 20ng/ml compared to 77.53% (138/178) of patients with PCa. Table 2 also shows that most of the PCa cases without prostatitis had higher PSA values when compared to PCa cases with prostatitis (Table 3). We found that in BPH with prostatitis, the median PSA was 15 ng/ml (IQR 9-24), while in BPH without prostatitis, the median PSA was 11ng/ml (IQR 7-16). We also found a statistically significant difference in the PSA value (Fischer's Exact test p < 0.017) between the two groups. This means that prostatitis increases PSA in patients with BPH.

**Table 3:** association between PSA, prostatitis in patients with BPH and PCa

	PSA ng/ml	4-10	11-20	21-50	51-100	Total	P value
BPH	Prostatitis	29	34	25	8	96	< 0.017
	No Prostatitis	31	27	6	3	67	
	Total	60	61	31	11	163	
PCa	Prostatitis	5	4	15	17	41	0.4
	No prostatitis	15	16	33	73	137	
	Total	20	20	48	90	178	

In patients with PCa and prostatitis, the median PSA was 44 ng/ml (IQR 21-100), whereas in patients with PCa with no prostatitis, the median PSA was 53 ng/ml (IQR 23-100). We did not find any statistically significant difference in the PSA values between the two groups (Fischer's exact test p=0.418). This suggests that the presence of prostatitis did not significantly influence the PSA value in patients with prostate cancer.

Table 4 demonstrates that most cases of prostatitis were chronic. Chronic prostatitis was identified in 89.87% (142/158) cases, followed equally by acute prostatitis and acute on chronic prostatitis with 5.06% each (8/158).

**Table 4:** distribution of histological types of prostatitis

	Histology finding	Frequency	Percentage
Chronic prostatitis	Chronic prostatitis	142	89.87
Acute prostatitis	Mild acute	4	2.53
	Moderate acute	2	1.27
	Severe acute	2	1.27
	Acute on chronic	8	5.06
	Total	158	100%

## Discussion

The mean age of patients with prostatic disease who underwent a prostate biopsy at Potchefstroom Hospital was 69.82±7.9 years. The mean age of patients with prostatitis was 70.79±7.69 years. Authors such as Edlin RS *et al*. and Van Vuuren SPJ *et al*. found similar mean ages of 67.2 years and 66.4 years respectively in South Africa [16,17]. In contrast, according to Rizzo M *et al*., patients with clinical symptoms of acute prostatitis presenting at the urologist were found to be younger, around 47 years [13]. Our mean age may differ as our sample was taken only from patients who had had prostate biopsies as a workup for prostate cancer, and these patients tend to be older.

The overall prevalence of prostatitis in this study was 43.65% (158/362). The subgroup analysis showed 89.87% chronic prostatitis (142/158). We found 5.06% (8/158) acute prostatitis and acute on chronic prostatitis. However, the prevalence of prostatitis alone (not associated with BPH or prostate cancer) was 5.8%, as shown in Table 1. A systematic review by Krieger *et al*. that looked at five studies from America and Asia, reported an overall prevalence of clinician-assigned prostatitis of 8.2%, with values ranging from 2.2% to 9.7% [1]. In a more recent systematic review, Polackwich *et al*. reported the prevalence of chronic prostatitis as 4.5% to 9% [6]. For many authors, the prevalence of clinically diagnosed prostatitis is reported below or around 10% [13,15,19,20].

Our data demonstrated a higher prevalence of prostatitis in patients with BPH, 58.9% (96/163), than prostatitis in patients with prostate cancer, 23.03% (41/178). Similarly, Edlin RS *et al*. found a higher prevalence of prostatitis associated with BPH than prostatitis associated with prostate cancer, 61.0% (130/213) compared to 29.7% (51/172) [16]. The difference was statistically significant (p < 0.001) [16]. Another study by Van Vuuren *et al*. looked at histological prostatitis in patients with urinary retention, which also demonstrated a higher prevalence of prostatitis associated with BPH, 48%, compared to prostate cancer, 25% [17]. These two studies are comparable to our study as they too considered histologically diagnosed prostatitis and were done in South Africa.

A study by M Collins *et al*. showed that patients with BPH were seven times more likely to have prostatitis [21]. Furthermore, a study by Lauren Wallner *et al*. found that men who had previously been diagnosed with BPH were eleven times more likely to have reported a history of prostatitis [19]. A recent systematic review and meta-analysis by Zhang *et al*. that examined the relationship between BPH and prostatitis and PCa and prostatitis, found an association between prostatitis and BPH with an odds ratio of 2.95 (CI=1.94-4.47) [7]. The association between prostatitis and PCa was also shown, but with an odds ratio of only 1.59 (CI=1.48-1.70) [7]. While this shows an association between both prostatitis and BPH, as well as prostatitis and PCa, the association between prostatitis and BPH is stronger. Our findings support the association between prostatitis and BPH, but do not demonstrate the association between prostatitis and the diagnosis of prostate cancer to be significant.

It is still unclear to what extent there is a connection between prostatitis and raised PSA levels, but leakage of PSA, altered vascular permeability, hyper-vascularity of the prostate, and an increase in cell death are possible pathways to explain high PSA values in prostatitis [22]. PSA can remain elevated up to 2 to 3 months after an acute episode, especially when associated with acute bacterial prostatitis [22]. We found a statistically significant association (p=0.02) between elevated PSA and the diagnosis of prostatitis when we compared the median PSA value between patients with BPH alone (PSA 11ng/ml, IQR 7-16) to patients with BPH associated with prostatitis (PSA 15ng/ml, IQR 9-24). In contrast, we did not find any statistically significant difference (p=0.42) in median PSA value in the PCa group with prostatitis (PSA 44 ng/ml, IQR 21-100) versus those without prostatitis (PSA 53ng/ml, IQR 23-100). Both Edlin RS *et al*. and Van Vuuren SPJ reached the same conclusion in their studies on the effect of histological prostatitis on the PSA value [16,17].

**Limitations of the study:** this is a retrospective study with some clinical information missing, therefore, we could not do an in-depth exploration of some topics like the association of prostatitis in patients with indwelling catheter. There were some variations in the way in which the histopathology was reported. This lack of standardisation did not allow us to regroup the prostatitis cases as per the NIH classification. The prostatitis cases reported in this study did not take in account the clinical symptoms and were solely based on the histopathology report. Current practice in biopsy techniques differs from the trans-perineal technique used at the research site. However, we do not believe that this would have significantly changed the outcome.

## Conclusion

This study showed that close to half of the histology reports examined showed that patients had signs of prostatitis. Chronic prostatitis was more frequent in histology done in our setting compared to acute prostatitis. BPH was strongly associated with prostatitis compared to PCa. Prostatitis contributed to higher PSA values in patients with BPH and did not influence the PSA value in PCa. The higher prevalence of prostatitis in histology in our setting calls for a cautious interpretation of the PSA value in the absence of the histology report.

### What is known about this topic

- *Prostatitis is a common urological condition in elderly patients;*
- *The histological prevalence is higher than reported clinically*.

### What this study adds

- *This is the first published study on the prevalence of prostatitis at a regional hospital in Potchefstroom, North West province, South Africa;*
- *The study shows that close to half of the patients above fifty years have prostatitis based on histology;*
- *This study contributes to the body of data available on the topic and could therefore influence further research in similar settings*.
